# Supplementary material for: Chemo-profiling of Purpureocillium lilacinum and Paecilomyces variotii isolates using GC-MS analysis, and evaluation of their metabolites against M. incognita
Source: PLoS One. 2024 Feb 15;19(2):e0297925. doi: 10.1371/journal.pone.0297925 (PMC10868743; doi:10.1371/journal.pone.0297925)
Supplement: S1 Table — (PDF) [file pone.0297925.s001.pdf]

Table S1 GenBank accession number of *P. lilacinum* and *P. variotii* isolates used in the study.

Isolate name with *ITCC accession number	GenBank accession
Paecilomyces variotii 0746	OQ147234
Paecilomyces variotii 1833	OQ147235
Purpureocillium lilacinum 2362	OQ147236
Purpureocillium lilacinum 4483	OQ147237
Purpureocillium lilacinum 4899	OQ147238
Purpureocillium lilacinum 4910	OQ147239
Purpureocillium lilacinum 5596	OQ147240
Purpureocillium lilacinum 6064	OQ147241
Purpureocillium lilacinum 6381	OQ147242
Purpureocillium lilacinum 6553	OQ147243
Purpureocillium lilacinum 6887	OQ147244
Purpureocillium lilacinum 6948	OQ147245
