# Supplementary figures and images for: Chemo-profiling of Purpureocillium lilacinum and Paecilomyces variotii isolates using GC-MS analysis, and evaluation of their metabolites against M. incognita
Source: PLoS One. 2024 Feb 15;19(2):e0297925. doi: 10.1371/journal.pone.0297925 (PMC10868743; doi:10.1371/journal.pone.0297925)

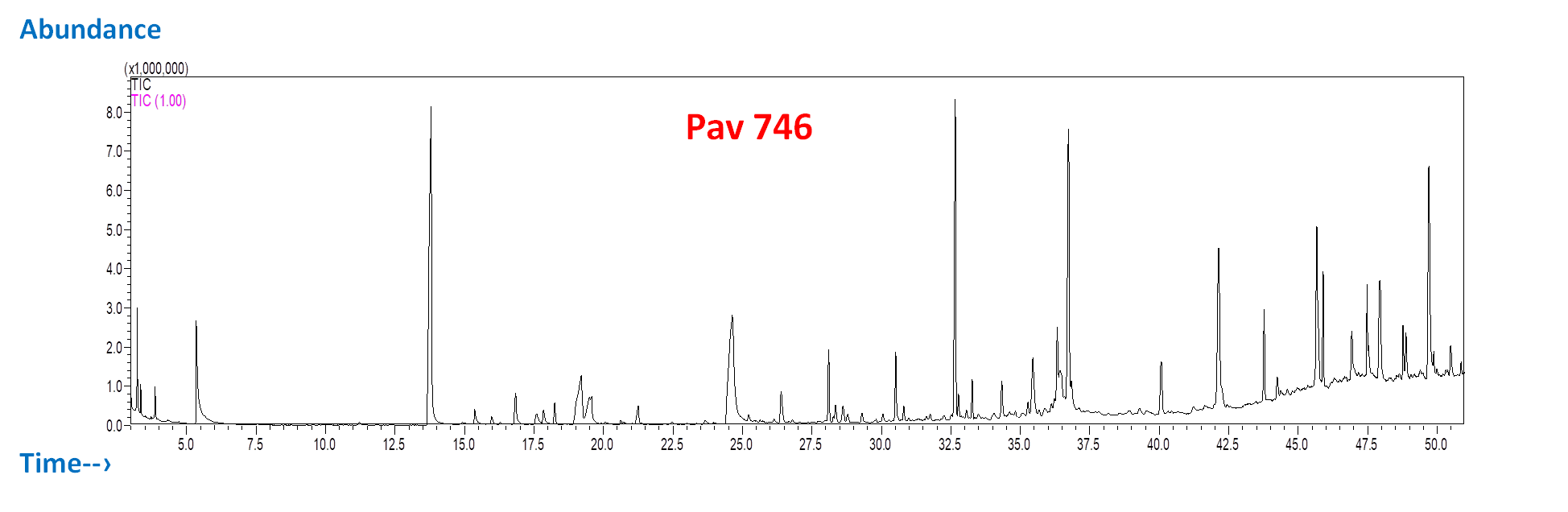

Supplement: S1 Fig — (PNG) [file pone.0297925.s002.png]

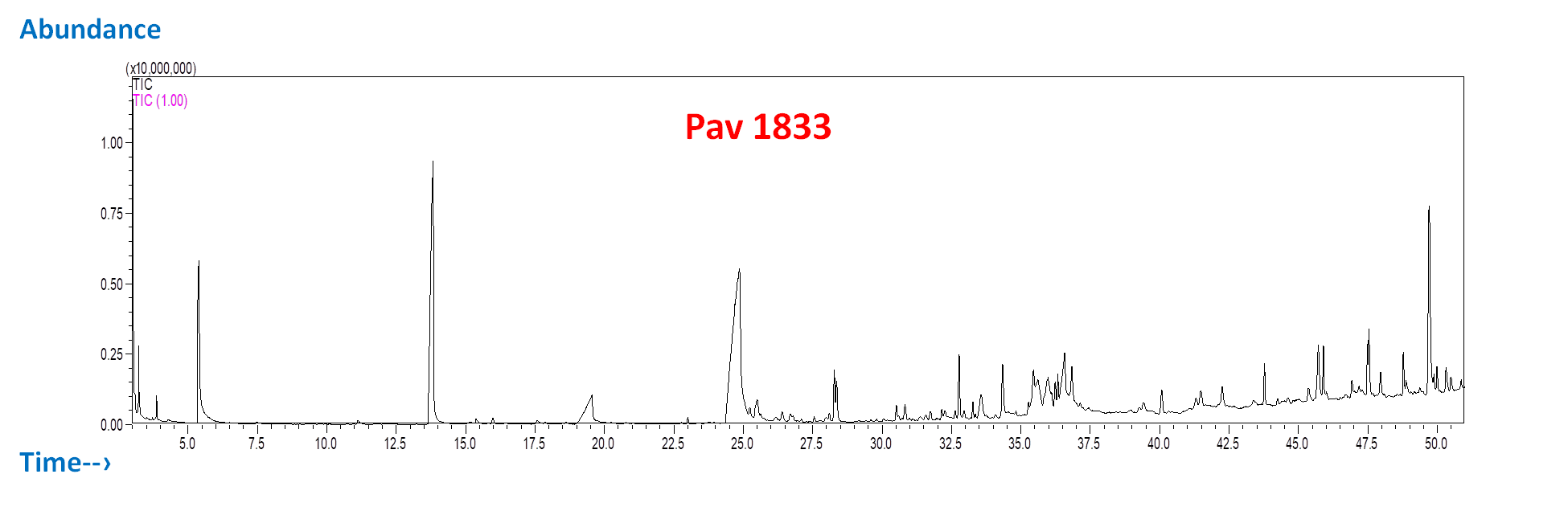

Supplement: S2 Fig — (PNG) [file pone.0297925.s003.png]

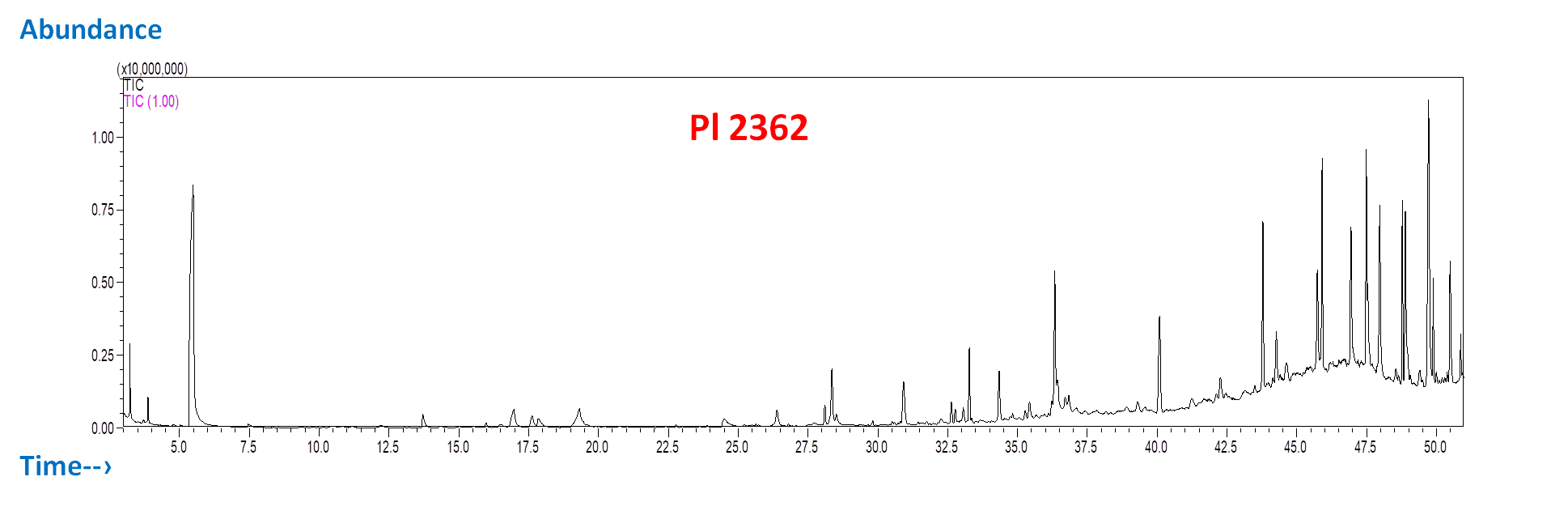

Supplement: S3 Fig — (PNG) [file pone.0297925.s004.png]

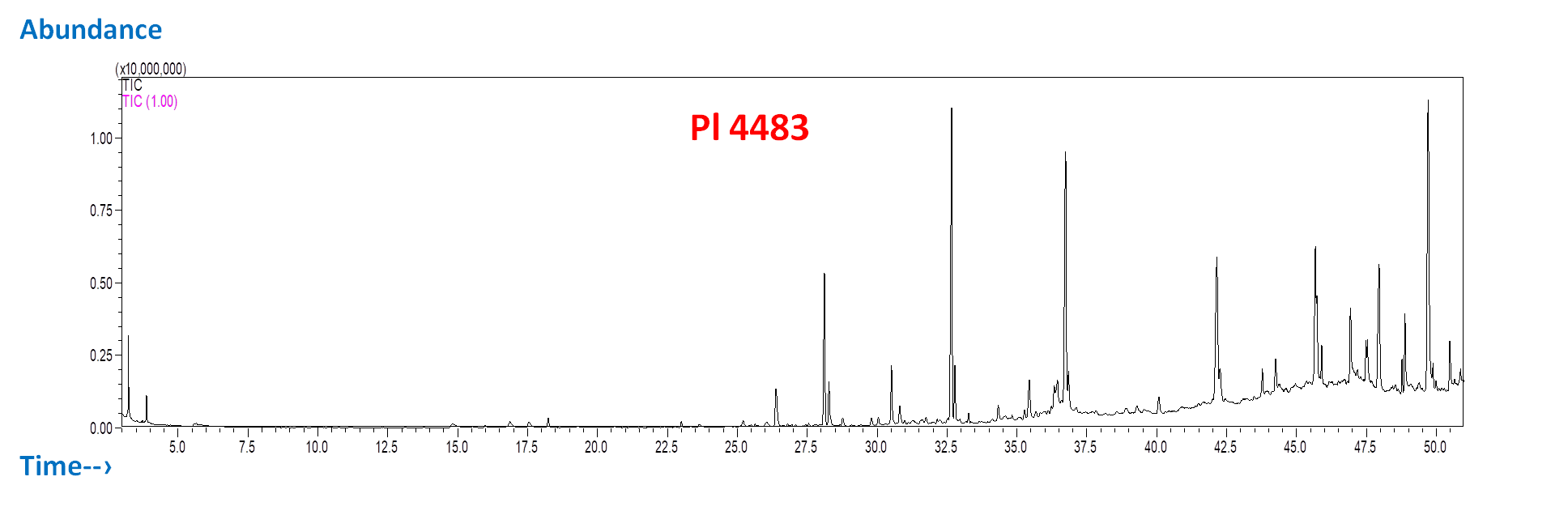

Supplement: S4 Fig — (PNG) [file pone.0297925.s005.png]

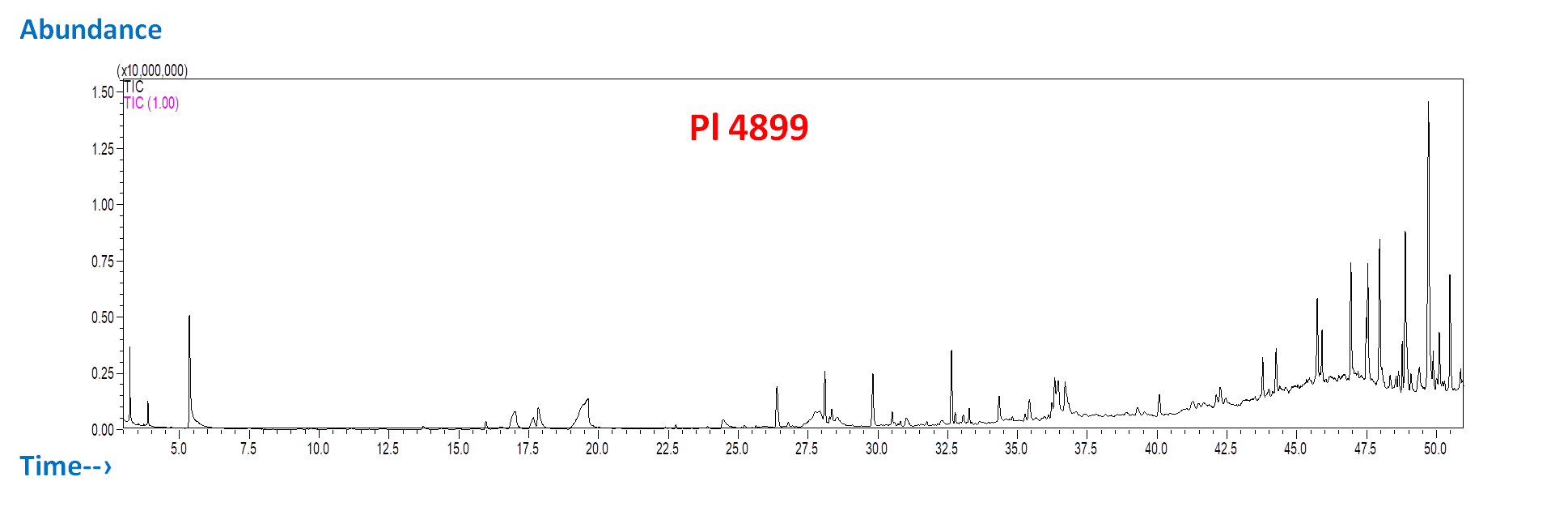

Supplement: S5 Fig — (PNG) [file pone.0297925.s006.png]

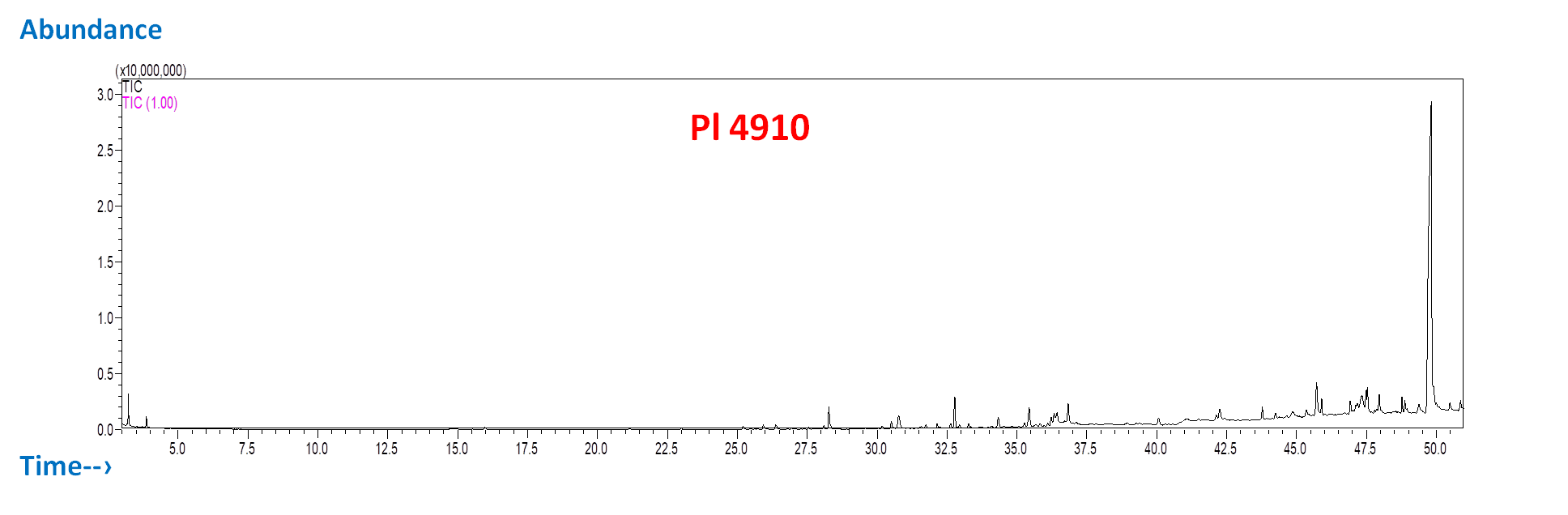

Supplement: S6 Fig — (PNG) [file pone.0297925.s007.png]

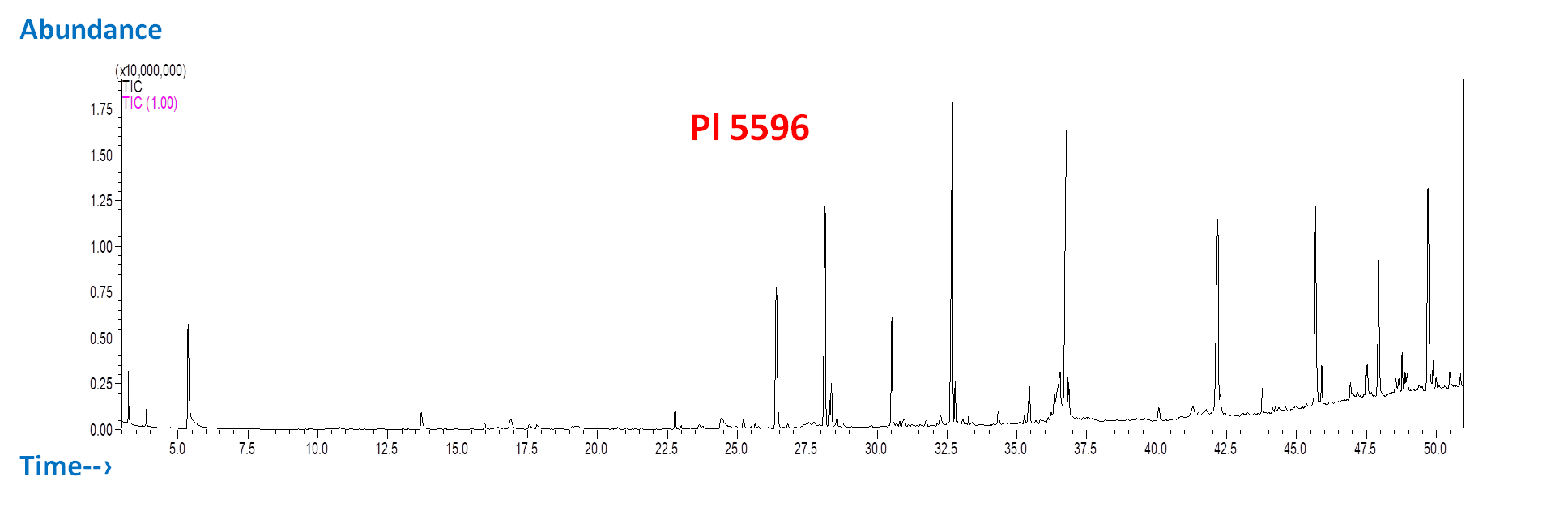

Supplement: S7 Fig — (PNG) [file pone.0297925.s008.png]

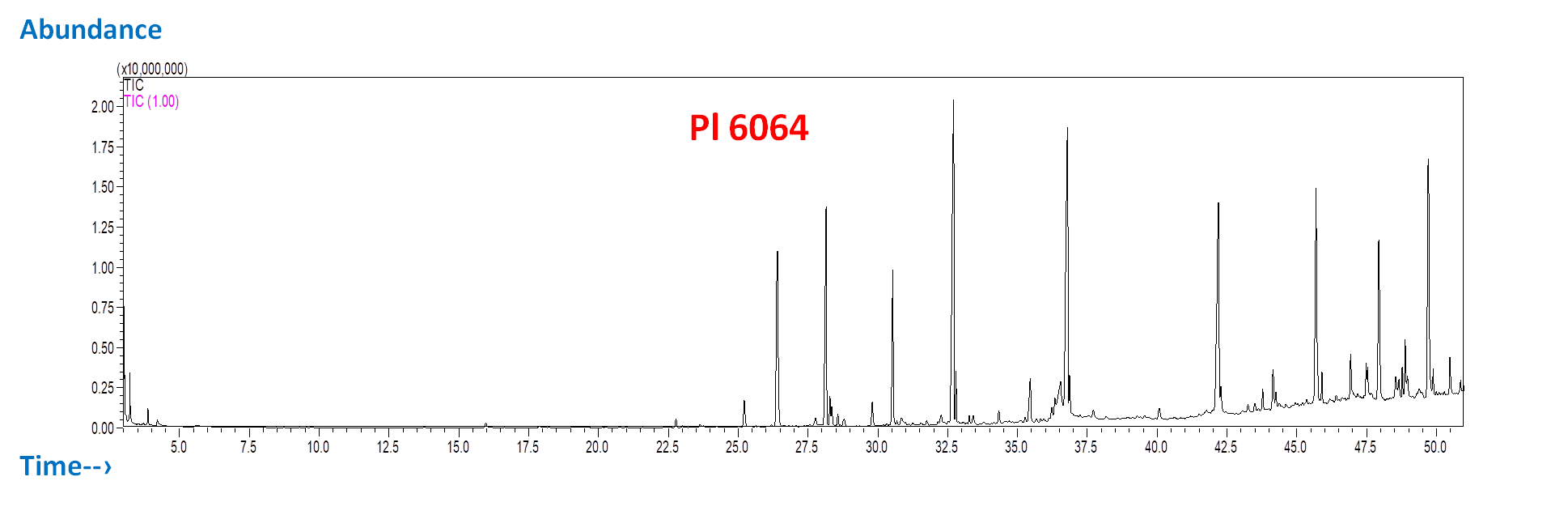

Supplement: S8 Fig — (PNG) [file pone.0297925.s009.png]

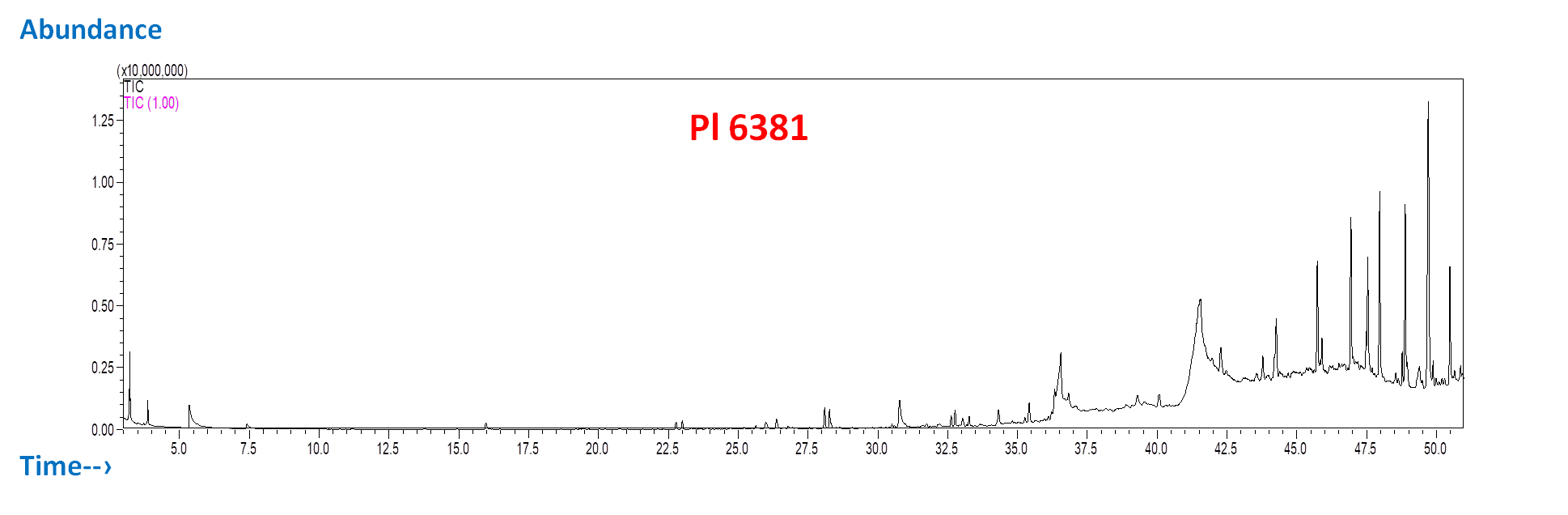

Supplement: S9 Fig — (PNG) [file pone.0297925.s010.png]

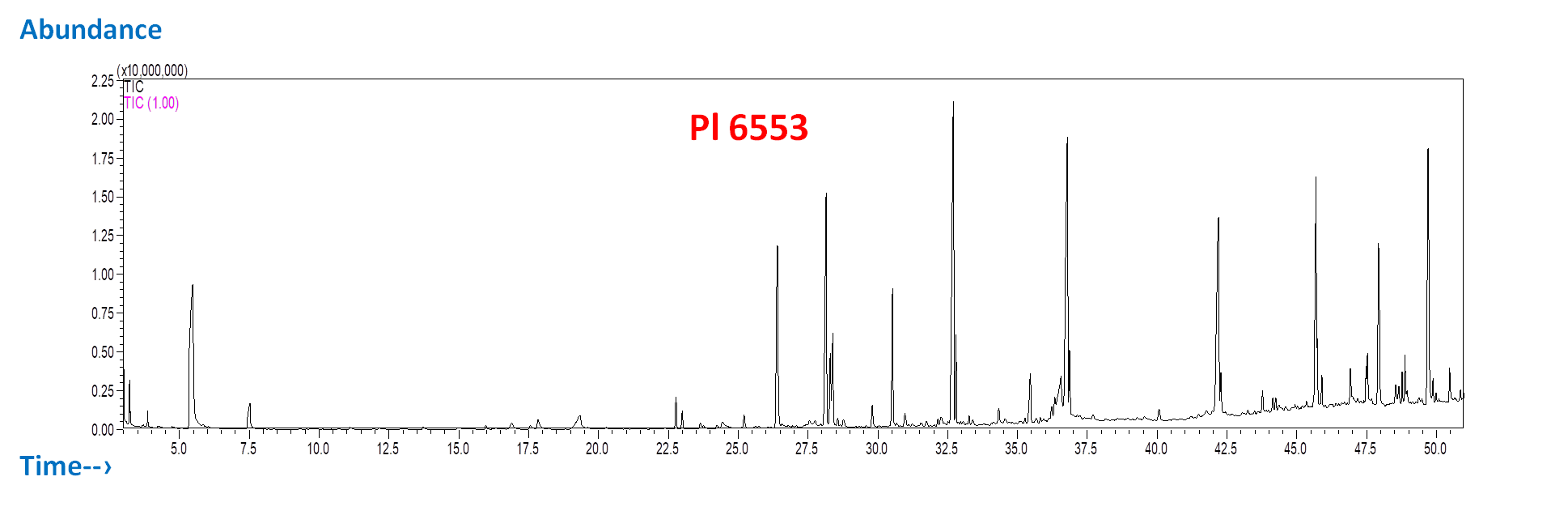

Supplement: S10 Fig — (PNG) [file pone.0297925.s011.png]

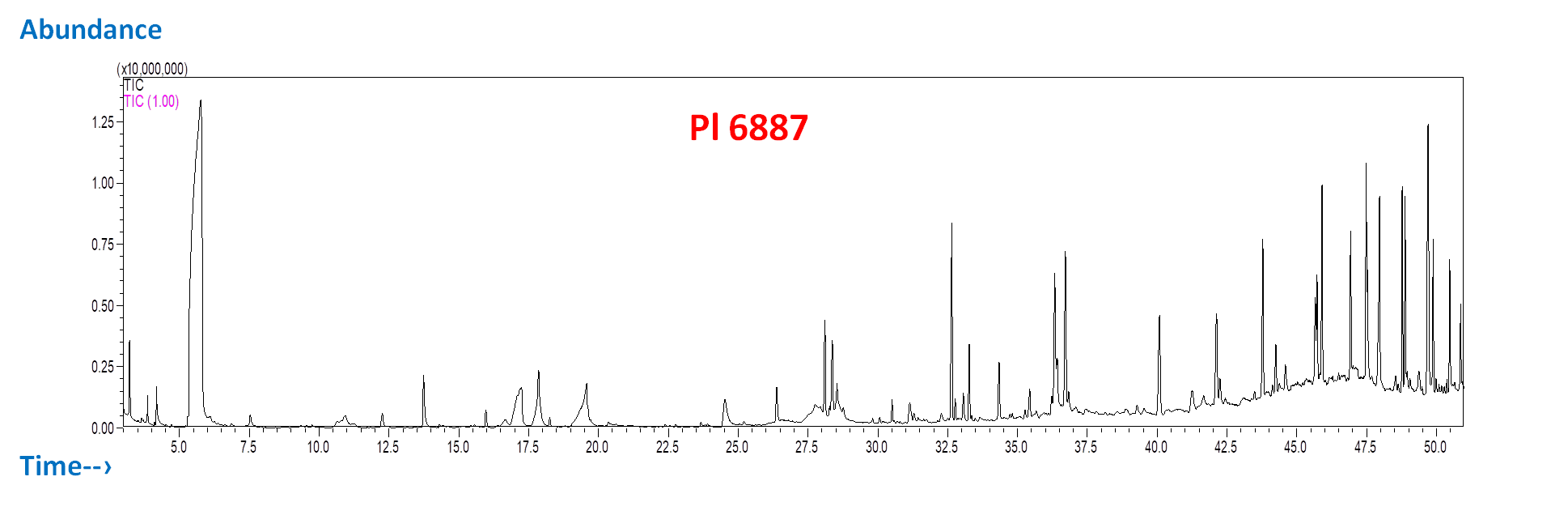

Supplement: S11 Fig — (PNG) [file pone.0297925.s012.png]

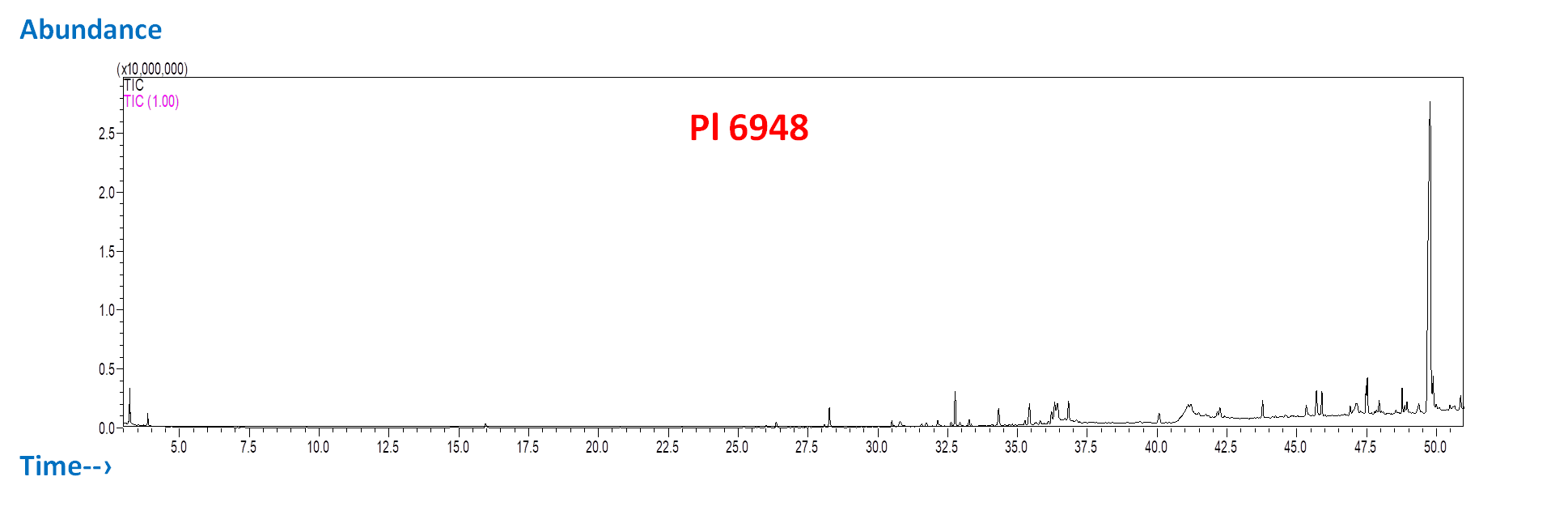

Supplement: S12 Fig — (PNG) [file pone.0297925.s013.png]
