# Supplementary material for: Chemo-profiling of Purpureocillium lilacinum and Paecilomyces variotii isolates using GC-MS analysis, and evaluation of their metabolites against M. incognita
Source: PLoS One. 2024 Feb 15;19(2):e0297925. doi: 10.1371/journal.pone.0297925 (PMC10868743; doi:10.1371/journal.pone.0297925)
Supplement: Attachment — Submitted filename: Paper Plos One.docx [file pone.0297925.s014.docx]

**Chemo-profiling of *Purpureocillium lilacinum* and *Paecilomyces variotii* isolates using GC-MS-NIST data and evaluation of their metabolites against *M. incognita***

**Prashant Patidar^1^, *Lakshman Prasad^1^, Sushma Sagar^1^, Anil Sirohi^2^, M.S. Saharan^1^, Mukesh Kumar Dhillon^3^, Vaibhav Kumar Singh^1^, Tusar Kanti Bag^1^**

**^1^Prashant Patidar**

Division of Plant Pathology, ICAR-Indian Agricultural Research Institute, New Delhi, India

Email: [Pp97545@gmail.com](mailto:Pp97545@gmail.com)

***^1^Lakshman Prasad**

Division of Plant Pathology, ICAR-Indian Agricultural Research Institute, New Delhi, India

Email: [laxmanprasad25@yahoo.com](mailto:laxmanprasad25@yahoo.com)

**^1^Sushma Sagar**

Division of Plant Pathology, ICAR-Indian Agricultural Research Institute, New Delhi, India

Email: [sagarlovy@gmail.com](mailto:sagarlovy@gmail.com)

**^1^Anil Sirohi**

Division of Nematology, ICAR-Indian Agricultural Research Institute, New Delhi, India

Email: [anilsirohi@yahoo.com](mailto:anilsirohi@yahoo.com)

**^1^Mukesh Kumar Dhillon**

Division of Entomology, ICAR-Indian Agricultural Research Institute, New Delhi, India

Email: [mukeshdhillon@rediffmail.com](mailto:mukeshdhillon@rediffmail.com)

**^1^Mahender Singh Saharan**

Division of Plant Pathology, ICAR-Indian Agricultural Research Institute, New Delhi, India

Email: [mssaharan7@gmail.com](mailto:mssaharan7@gmail.com)

**^1^Vaibhav K Singh**

Division of Plant Pathology, ICAR-Indian Agricultural Research Institute, New Delhi, India

Email: [vaibhav.singh@icar.gov.in](mailto:vaibhav.singh@icar.gov.in)

**^1^Tusar Kanti Bag**

Division of Plant Pathology, ICAR-Indian Agricultural Research Institute, New Delhi, India

Email:  [tusar.bag@icar.gov.in](mailto:tusar.bag@icar.gov.in)

***Corresponding author email:** [laxmanprasad25@yahoo.com](mailto:laxmanprasad25@yahoo.com)

**Abstract**

Nematophagous fungi are the best alternatives to chemical nematicides for managing nematodes, considering environmental health. In the current study, metabolites of ten *Purpureocillium lilacinum* (Thom) Luangsa-ard (Hypocreales: Ophiocordycipitaceae), and two *Paecilomyces variotii* Bainier (Eurotiales: Trichocomaceae) isolates were screened for their ability to inhibit *Meloidogyne incognita* (Kofoid & White) Chitwood (Tylenchida: Heteroderidae) egg hatching. Culture filtrate of the isolate *P. lilacinum* 6887 inhibited 97.55%, 90.52%, and 62.97% of egg hatching at 100%, 50% and 25% concentrations, respectively. The isolates, namely Pl 6887, Pl 6553 and Pl 2362 performed best in the hatching inhibition assay among all the isolates. Gas chromatography-mass spectrometry analysis revealed a variety of nematicidal compounds from different isolates. A total of seven nematicidal compounds, including four very potent nematicidal fatty acids were found in the isolate Pl 6553. Secondary metabolites of the same isolate possess the highest *M. incognita* juvenile mortality, i.e., 43.33% and 92% after 48 hrs of treatment at 100 and 200 ppm concentrations, respectively. Nematicidal fatty acids like myristic and lauric acid were found for the first time in *P. lilacinum*. Unhatched eggs in culture filtrate of isolate Pl 6887, and juveniles died in the ethyl acetate extract of isolate Pl 6553 had multiple vacuoles-like droplets inside.

**Keywords: Root-knot nematode, *Meloidogyne incognita*, *Purpureocillium lilacinum*, *Paecilomyces variotii*, Hatching inhibition, Nematicidal compounds**

**Introduction**

Plant parasitic nematodes (PPNs) are potential threats to crop production. Global loss due to PPNs is estimated to be $157 billion annually (Abad et al. 2008). Although the number of plant-parasitic nematode species identified is over 4,100, undoubtedly, the most yield-reducing group is the root-knot nematode (*Meloidogyne* spp.). It ranks top in terms of agricultural damage globally (Jones et al. 2013). Out of the 101 RKN species described, *Meloidogyne incognita* is the most economically important species in the tropical and subtropical regions (Pulavarty et al. 2021; Eisenback 2020). Along with the direct damage, an association of RKN with fungal and bacterial plant pathogens is common and results in disease complexes, causing more damage to the crops than the nematode alone (Mai and Abawi 1987). Chemical nematicides are widely used to control RKNs worldwide. However, due to their adverse effects on humans, environmental health, and non-targets, the use of chemical nematicides is not feasible in the current scenario of eco-friendly crop-pest management (Peiris et al. 2020). It results in the phase-out of several popular nematicides from the market (Desaeger et al. 2020). Hence, biological pesticides are becoming an important component of eco-friendly management systems (Davies and Spiegel, 2011a). *Purpureocillium lilacinum* (earlier *Paecilomyces lilacinus*) is one of the various biocontrol agents of the cyst and root-knot nematodes and is found most effective as an egg and female parasite (Siddiqui and Mahmood 1996). It is considered one of the most promising and practicable biological control agents for the management of sedentary endoparasitic PPNs (Morgan-Jones et al. 1984) and has been used successfully to manage root-knot nematodes in various crop plants (Jatala, 1986; Hewlett et al. 1988; Siddiqui and Mahmood 1996, Jacobs 2002; Kiewnick and Sikora 2006; Huang et al. 2016). Nematicidal metabolites secreted by *P. lilacinum* during infection of RKNs make its job even easier, and studies suggest that potential isolates of *P. lilacinum* are efficient producers of these metabolites (Park et al. 2004; Teles and Takahashi 2013; Sharma et al. 2020). Similarly, *Paecilomyces variotii* has also been reported to produce compounds of agricultural importance, including some nematicidal ones (Degenkolb and Vilcinskas 2015).

The present study aimed at determining the efficacy of ten *P. lilacinum* and two *Paecilomyces variotii* isolates against *M. incognita, in vitro,* as well as studying their secondary metabolites in nematode control. We found significant variation in the hatching inhibition obtained from the culture filtrate of *P. lilacinum* and *Paecilomyces variotii* isolates. Along with that, GC-MS analysis of metabolites of these isolates revealed a range of nematicidal compounds, while a couple of them are reported for the first time. The presence of a higher number of nematicidal compounds correlated with the mortality of *M. incognita* juveniles.

**Materials and methods**

**Fungal cultures**

For this study, ten isolates of *Purpureocillium lilacinum* and two isolates of *Paecilomyces variotii* were obtained from the Indian Type Culture Collection (ITCC), Division of Plant Pathology, ICAR-Indian Agricultural Research Institute, New Delhi. The isolates were maintained at 25 °C on PDA media.

**Cultural and morphological identification of the isolates**

Investigation on morphological characters was done using a compound microscope (ZEISS) with a camera attached to it. Morphological identification of the isolates was done based on the earlier diagnostic keys and descriptions of the species (Luangsa-ard et al. 2004, 2011).

**Molecular identification of the isolates**

The fungal DNA of all 12 isolates was extracted using the CTAB method from seven-day-old mycelia. The ITS region was amplified using the ITS primers (primer sequences: ITS1: 5' TCCGTAGGTGAACCTGCGG 3' and ITS4: 5' TCCTCCGCTTATTGATATGC 3') and PCR was performed. Confirmation of PCR amplification was done with the help of agarose gel electrophoresis. The amplified DNA was sequenced using Sanger sequencing and the obtained sequences were compared with the reference sequences available in the NCBI database to identify the species based on sequence similarities (blast.ncbi.nlm.nih.gov/). Phylogenetic analysis was performed using MEGA-X software with the Maximum Likelihood method. The Jukes-Cantor model was used for tree construction by keeping 1000 bootstrap replications.

***In vitro* egg hatching inhibition assay**

*Preparation of M. incognita inoculum*

Pure culture of *Meloidogyne incognita* was maintained on tomato (*Solanum lycopersicum* Mill. cv. Pusa ruby) grown in earthen pots filled with steam-sterilized sand and soil mixtures. The egg masses were separated from the roots with the help of a needle, while eggs were separated from the egg masses using a 1% sodium hypochlorite (NaOCl) solution (Ehwaeti et al. 1998). The eggs were passed through a 100-mesh sieve nested over 200 and 500-mesh sieves. The eggs collected over a 500-mesh sieve were gently rinsed with sterile distilled water to remove NaOCl residue and decanted in a clean sterile beaker. The egg-hatching inhibition assay was performed on freshly extracted eggs.

*Egg-hatching inhibition assay*

All twelve isolates were grown in a 500 ml Erlenmeyer flask containing 300 ml of sterilized potato dextrose broth (HiMedia), followed by incubation at 25°C for 21 days. The culture filtrate of the isolates was obtained by filtering through a 0.22 µm millipore filter to get a cell-free filtrate.

In a 24-well ELISA plate, a 20 µl suspension of *M. incognita* eggs containing 100 eggs was pipetted in each well. The culture filtrate of the isolates was added into the wells at three different concentrations, that is, 100%, 50%, and 25% with 4 replications each. Sterile distilled water was taken as control. At 50 and 25% concentrations of the culture filtrate tested, the volume was made up to 1 mL by adding sterile distilled water. The ELISA plates were incubated at 27 °C in a BOD incubator after sealing them with parafilm to reduce evaporation. The number of hatched juveniles was monitored regularly, while the final counting of the hatched and unhatched eggs was done on the 7th day of inoculation using a phase contrast microscope. The egg hatching inhibition in the treatments was calculated by the formula HI % = (Total number of hatched J2 in control – Number of hatched J2 in the treatment/ Total number of hatched J2 in control) × 100 (Kesba et al. 2021), which also eliminates the natural mortality in the treatments.

***GC-MS analysis***

The culture filtrate of each isolate was extracted thrice, with an equal volume of ethyl acetate. The aqueous phase was concentrated using a rotary evaporator (Heidolph, Germany) at reduced pressure and a temperature of 35 °C. The concentrated, dried metabolites were weighed and re-dissolved in GC-MS grade ethyl acetate at the rate of 1gm per ml. The GC-MS analysis was performed as described by Kassam et al. (2022) using Shimadzu GC-MS-QP 2010 using Rtx-1 MS column (30 m × 0.25 mm; 0.25 µm i.d.,) connected directly with a triple axis mass spectrometer. The injected sample volume was 1 µl. Helium was used as a carrier gas with a head pressure of 10 psi and flow of 0.75 mL min^−1^. The oven temperature was held initially at 40 °C and increased 4 °C min^−1^ to reach 120 °C. Then, the temperature increased again at the rate of 5 °C min^−1^ up to 200 °C with a hold time of 5 min. Finally, the temperature was raised to 300 °C with an increment of 10 °C min^−1^. The total run time was 51 min. The MS acquisition parameters were set with the ion source temperature 250 °C, electron ionization 60 eV, full scan mode (50–550 AMU), transfer line temperature 280 °C, and E.M voltage 1222. NIST (National Institute of Standards and Technologies) Mass Spectra Library was used for the identification of compounds by matching their mass spectra (Shimadzu Corporation, Kyoto, Japan).

**Juvenile mortality assay**

The juvenile mortality assay was conducted using extracted secondary metabolites of three *P. lilacinum* isolates Pl 6553, Pl 6887 and Pl 4483. It was found that these isolates had seven, five, and one compound, respectively, that were already reported as nematicidal.

**Preparation of stock concentration of secondary metabolites**

The dried secondary metabolites of the isolates Pl 6553, Pl 6887 and Pl 4483 were weighed and re-dissolved in methanol (1mg/ml) to make 1000 ppm stock concentration.

**Collection of *M. incognita* juveniles**

Tomato plants infected with *M. incognita* were uprooted, and the roots were washed free of soil with tap water. The individual egg masses were picked from the roots with the help of a toothpick and surface sterilized with 0.1% NaOCl. The egg masses were then kept in a clean Petri plate, and sterile distilled water was added to the plate just to cover the surface of the egg masses. The plates were kept at room temperature to allow the hatching of eggs. The freshly hatched juveniles were collected using a micropipette in a clean beaker. Counting juveniles per ml was done with the help of a stereomicroscope aided by a hand tally counter. Finally, the number of juveniles was adjusted to 100/ 20 µl suspension.

**The juvenile mortality assay**

For the juvenile mortality assay, two concentrations (100 and 200 ppm) of the methanol-dissolved secondary metabolites of the three *P. lilacinum* isolates (Pl 6553, Pl 6887 and Pl 4483) were used. ELISA plates, with 24 wells, were used for the bioassay. Each well received 100 *M. incognita* J2 (20 µl suspension), 10 µl (for 100 ppm concentration) or 20 µl (for 200 ppm concentration) methanol dissolved secondary metabolites of the isolates from the stock concentration of 1000 ppm. The volume was made up to 1 ml with 0.5% Tween 80. Each treatment was replicated three times and both 10 and 20 µl of pure methanol dissolved in 0.5% Tween 80, along with 100 *M. incognita* juveniles was taken as control. The plates were incubated at 27 °C in a BOD incubator after sealing them with parafilm. After 24 and 48 hours of incubation, the live and dead juveniles were counted. The freely moving juveniles were considered alive, whereas the juveniles, being straight and did not show any movement after probing with a nematode handling needle, were considered dead.

**Data analysis**

All percentage data were first transformed into arcsine value and then used for two-way Analysis of Variance (ANOVA) for hatching inhibition assay and three-way Analysis of Variance (ANOVA) for juvenile mortality assay. The post hoc multiple comparisons were done at a 5% significant level by Duncan’s Multiple Range Test (DMRT). All the analysis was carried out using OPSTAT software (<http://14.139.232.166/opstat/>).

**Results**

**Cultural and morphological identification of the isolates**

The ten isolates of *P. lilacinum* were identified as *Purpureocillium lilacinum* using the reference keys (Luangsa-ard et al. 2011). The colony colour on PDA media for all the isolates of *P. lilacinum* was lilac to vinaceous, which is typical of this fungus. Whereas morphological investigations revealed the production of phialides with long tapering necks, and ellipsoidal to fusiform conidia, varied from 2.6–3.40 × 2–2.8 µm in size. Both isolates of *Paecilomyces variotii* were matched with the keys (Samson 1974, Luangsa-ard et al. 2004) as they produced a white fluffy colony, which turns yellowish brown or sand colored during conidiogenesis. They also produced fusoid to broadly ellipsoid conidia, varied from 3.3–4.8 × 2–2.4 µm in size with visible multiple nuclei inside. Chlamydospores were observed only in one isolate (Pav 746), among the two isolates of *Paecilomyces variotii* studied.

**Molecular identification of the isolates**

After sequencing of PCR products of all the 12 isolates, the obtained sequences were BLAST on the NCBI database, which confirms ten *P. lilacinum* isolates and two *P. variotii* isolates. The GenBank accession number of all the isolates is mentioned in the supplementary table (Table S1). The phylogenetic tree (Fig. 1) among the isolates was constructed using the maximum likelihood method, which shows the close relatedness of *P. lilacinum* and *P. variotii* isolates with already available sequences of *P. lilacinum* and *Paecilomyces variotii*, respectively, on the NCBI database.

**Fig. 1 Phylogenetic relationship between *Purpureocillium lilacinum* and *Paecilomyces variotii* isolates based on ITS sequences constructed using the Maximum likelihood method**. The bootstrap values are indicated next to the tree branches and are based on 1000 bootsrap replications. Values in % are data coverage, and in decimal points are branch length.

**Hatching inhibition assay**

Significant variation in hatching inhibition was found among the treatments as compared to the control (Table 1). The isolate, Pl 6887 was the best-performing isolate, as the highest hatching inhibition (97.55%, 90.52%, and 62.97%) was achieved at 100%, 50%, and 25% concentration, respectively. It was the only isolate where more than 50% hatching inhibition was observed at the lowest concentration (25%) tested. However, hatching inhibition in the culture filtrate of the isolates Pl 6553 was comparable to Pl 6887 at 100% and 50% concentrations. The culture filtrate of the *Paecilomyces variotii* isolate Pav 1833, showed 90.52%, 56.88%, and 28.74% hatching inhibition at 100, 50, and 25% concentrations, respectively. The result obtained was better than three isolates of *P. lilacinum* (Pl 4483, Pl 5596 and Pl 6948). However, hatching inhibition in another *Paecilomyces variotii* isolate (Pav 746) was the lowest among all the isolates at 100% and 25% concentrations. Unhatched eggs of *M. incognita* from the culture filtrate of the isolates, Pl 6887 and Pl 6553, were deformed and vacuolated (Fig. 2) when visualized under a phase contrast microscope at higher magnification.

**Fig. 2** Effect of culture filtrate of *P. lilacinum* on *M. incognita* eggs. (a) Control, where the egg is under embryogenesis, and (b) egg with a giant vacuole (red arrow), two vacuoles fusing (white arrow) and multiple variously sized vacuoles like droplets in the culture filtrate of *P. lilacinum* isolate Pl 6887, at 7^th^ day of incubation. Scale bars of a and b were 50 μm.

**Table 1** List of isolates *Paecilomyces variotii* and *P. lilacinum* and effect of their culture filtrates on *M. incognita* egg hatching

| *Treatments | Name of Isolates | Hatching inhibition (%) | | | Means |
| --- | --- | --- | --- | --- | --- |
|  |  | 100% conc. | 50% conc. | 25% conc. |  |
| T1 | **Pav 746 | ^†^84.40 ^‡^(66.78)^f^**^***^**^*^ | 48.93 (44.39)^g^ | 10.71 (19.03)^g^ | 48.01(43.38) |
| T2 | Pav 1833 | 90.52 (72.14)^cd^ | 56.88 (48.96)^f^ | 28.75 (32.4)^de^ | 58.71(51.14) |
| T3 | ***Pl 2362 | 95.11 (77.35)^b^ | 73.70 (59.16)^cd^ | 37.92 (37.99)^c^ | 68.91(58.14) |
| T4 | Pl 4483 | 87.16 (69.03)^ef^ | 42.51 (40.68)^h^ | 12.54 (20.6)^g^ | 47.40(43.41) |
| T5 | Pl 4899 | 92.05 (73.68)^c^ | 75.54 (60.36)^c^ | 35.17 (36.36)^c^ | 67.58(56.77) |
| T6 | Pl 4910 | 91.13 (72.7)^c^ | 67.28 (55.12)^e^ | 30.89 (33.75)^d^ | 63.09(53.83) |
| T7 | Pl 5596 | 85.63 (67.73)^ef^ | 19.57 (26.22)^i^ | 11.93 (20.11)^g^ | 39.04(38.01) |
| T8 | Pl 6064 | 94.19 (76.1)^b^ | 70.95 (57.39)^d^ | 17.74 (24.85)^f^ | 60.95(52.75) |
| T9 | Pl 6381 | 94.80 (76.92)^b^ | 80.73 (63.97)^b^ | 17.13 (24.42)^f^ | 64.22(55.08) |
| T10 | Pl 6553 | 96.94 (79.98)^a^ | 89.30 (70.91)^a^ | 44.96 (42.1)^b^ | 77.06(64.30) |
| T11 | Pl 6887 | 97.56 (81.31)^a^ | 90.52 (72.09)^a^ | 62.97 (52.53)^a^ | 83.68(68.61) |
| T12 | Pl 6948 | 88.38 (70.1)^de^ | 40.67 (39.62)^h^ | 26.00 (30.63)^e^ | 51.68(46.76) |
| T13 | Control | 0.00 (0)^g^ | 0.00 (0)^j^ | 0.00 (0)^h^ | 0.00(00.00) |
| CD @p <0.05 | A (Isolates) | 1.300 | | | |
|  | B(Concentration) | 0.625 | | | |
|  | AxB | 2.252 | | | |

*Fungal culture filtrate and 100 eggs of *M. incognita*, **Pav=*Paecilomyces variotii*, ***Pl= *Purpureocillium lilacinum.*

^†^ Mean of 4 replications in percent; ^‡^ Arcsine transformation values in parentheses.

****Values in the same column, followed by common letter(s) are statistically not different as per Duncan’s multiple range test at 5% level of significance (p<0.05).

**Chemo-profiling analysis using Gas Chromatography Mass Spectroscopy**

GC-MS analysis of the ethyl acetate extract of all the 12 isolates revealed a large number of bioactive compounds. However, as of our interest, the compounds found as nematicidal in earlier studies (Dijan et al. 1991; Stadler et al. 1994; Park et al. 2004: Gapasin et al. 2011, Zhang et al. 2012, Teles and Takahashi 2013, Sharma et al. 2014c, Sharma et al. 2020 and Liu et al. 2022) were shortlisted (Table 2). The highest number of nematicidal compounds were found in Pl 6553 (7), followed by Pl 6887 (5). Nematicidal fatty acids, namely, lauric, myristic, stearic and butyric acid were found in Pl 6553 isolate, along with other nematicidal compounds like benzene acetic acid, 4-hydroxy benzene ethanol and benzoic acid. The only nematicidal compound found in Pl 4483 isolate was palmitic acid. The percent area of phenyl ethyl alcohol and 4-hydroxy benzene ethanol was much higher in the isolates of *Paecilomyces variotii* (Pav 746 and Pav 1833), as compared to the isolates of *P. lilacinum*. No nematicidal fatty acid, other than palmitic acid, was found in the ethyl acetate extract of *Paecilomyces variotii* isolates.

**The juvenile mortality assay**

The ethyl acetate extract of all three *P. lilacinum* isolates showed differences in the mortality of *M. incognita* juveniles (Table 3). The isolate Pl 6553 showed the highest mortality of the juveniles i.e., 11.66% and 43.33% at 100 ppm, and 25% and 92% at 200 ppm concentrations, after 24 and 48 hours of treatment, respectively. While the lowest juvenile mortality among

**Table 2** List of nematicidal compounds identified using GC-MS analysis in *Paecilomyces variotii* and *P. lilacinum* isolates

| Nematicidal compounds | Molecular formula | RT* in minute | Retention area (%) | | | | | | | | | | | | References**** |
| --- | --- | --- | --- | --- | --- | --- | --- | --- | --- | --- | --- | --- | --- | --- | --- |
|  |  |  | Pav** 746 | Pav 1833 | Pl*** 2362 | Pl 4483 | Pl 4899 | Pl 4910 | Pl 5596 | Pl 6064 | Pl 6381 | Pl 6553 | Pl 6887 | Pl 6948 | Sharma et al. 2020 |
| Phenyl ethyl alcohol | C_8_H_10_O | 13.76 | 25.40 | 20.93 | 1.56 | — | — | — | 1.78 | — | — | — | 3.66 | — | Dijan et al. 1991 |
| Acetic acid | CH₃COOH | 18.61 | — | — | — | — | — | — | — | — | — | — | 5.32 | — | Sharma et al. 2020 |
| Benzene acetic acid | C_8_H_8_O_2_ | 19.54 | — | 5.44 | 4.11 | — | — | — | — | — | — | 1.25 | — | — | Sharma et al. 2020 |
| 4-hydroxy benzene ethanol | C_8_H_12_O_2_ | 24.53 | 15.47 | 29.27 | — | — | 3.52 | — | 3.63 | — | — | 1.75 | 6.40 | — | Stadler et al. 1994 |
| Lauric acid | C_12_H_24_O_2_ | 27.71 | — | — | — | — | — | — | — | 0.30 | — | 0.11 | — | — | Sharma et al. 2020 |
| Benzoic acid | C_7_H_6_O_2_ | 33.05 | 0.18 | — | 0.46 | — | 0.37 | — | 0.24 | 0.09 | — | 0.14 | — | — | Stadler et al. 1994; Zhang et al. 2012 |
| Myristic acid | C_14_H_28_O_2_ | 32.25 | — | — | — | — | — | — | — | 0.18 | — | 0.16 | — | — | Stadler et al. 1994; Zhang et al. 2012; |
| Palmitic acid | C_16_H_32_O_2_ | 36.45 | 0.77 | 1.49 | 1.50 | 1.60 | 1.89 | 0.76 | — | 1.53 | 3.98 | — | 1.65 | 1.43 | Stadler et al. 1994; Zhang et al. 2012; Teles and Takahashi 2013; Sharma et al. 2014c |
| Oleic acid | C_18_H_34_O_2_ | 41.10 | — | — | — | — | 0.05 | 0.14 | 0.04 | — | — | — | — | 0.11 | Stadler et al. 1993a; Stadler et al. 1994; Zhang et al. 2012; Teles and Takahashi 2013; Sharma et al. 2014c |
| Linoleic acid | C_18_H_32_O_2_ | 41.47 | — | — | — | — | — | — | — | — | 1.62 | — | — | 0.62 | Stadler et al. 1994; Zhang et al. 2012; Teles and Takahashi 2013; Sharma et al. 2014c |
| Stearic acid | C_18_H_36_O_2_ | 41.77 | — | — | — | — | — | — | 0.09 | — | 0.20 | 0.22 | 0.15 | — | Zhang et al. 2012; Sharma et al. 2014c; Browning et al. 2004; Browning et al. 2006; Ntalli et al. 2010 |
| Butyric acid | C_4_H_8_O_2_ | 48.95 | — | — | — | — | — | 0.48 | 0.74 | 0.85 | — | 0.43 | — | — |  |

**Note**: ‘—‘means not detected. * the compounds are listed based on their retention time in minute, ***Paecilomyces variotii*, *** *Purpureocillium lilacinum*

****References where these compounds are reported as nematicidal and not necessarily secreted by *P. lilacinum* or *Paecilomyces variorti*

The isolate was observed with Pl 4483 i.e., 4% and 10% at 100 ppm, and 10% and 18.66% at 200 ppm, after 24 and 48 hours of treatment, respectively. In GC-MS analysis, it was found that the earlier isolate has seven reported nematicidal compounds, while the latter has only one. The juvenile mortality observed in the Pl 6887 isolate was in between the two other isolates and so was the number of nematicidal compounds. Multiple vacuoles were observed in the juveniles after 48 hours of incubation in the metabolites of Pl 6553 isolate (Fig. 3b), while the juveniles in control were freely moving and lacking any vacuoles inside the body (Fig. 3a).

**Fig. 3** *M. incognita* juvenile after 48 hours of incubation in (A) control having stylet intact (more clear in inset, Bar 10 μm) and (B) in *P. lilacinum* isolate Pl 6553 metabolites having degenerated head region (more clear in inset) and with vacuoles-like droplets (black arrow). Scale bars of a and b was 50 μm while 10 μm in insets.

**Table 3:** Effect of *P. lilacinum* metabolites on *M. incognita* juvenile mortality.

| Treatments | Isolates | % Juvenile mortality | | | |
| --- | --- | --- | --- | --- | --- |
|  |  | 100 ppm concentration | | 200 ppm concentration | |
|  |  | 24 h | 48 h | 24 h | 48 h |
| T1 | *Pl 6553 | ^†^11.66 (^‡^19.94) ^a^**^***^** | 43.33 (41.17)^a^ | 25.00 (29.99)^a^ | 92.00 (73.59)^a^ |
| T2 | Pl 6887 | 8.66 (17.12) ^b^ | 27.33 (31.52)^b^ | 14.66 (22.50)^b^ | 57.00 (49.02)^b^ |
| T3 | Pl 4483 | 4.00 (11.48) ^c^ | 10.00 (18.42)^c^ | 10.00 (18.42)^c^ | 18.66 (25.52)^c^ |
| T4 | **Control | 2.33 (8.74) ^d^ | 5.66 (13.76)^d^ | 3.33 (10.49)^d^ | 7.33 (15.70)^d^ |
| CD @p <0.05 | A (Isolates) | 1.027 | | | |
|  | B (Concentration) | 0.726 | | | |
|  | C (Time interval) | 0.726 | | | |
|  | AxBxC | 2.054 | | | |

*Pl=*Purureocillium lilacinum*

**Control= (1% Methanol in 0.5% tween 80 + 100 J2 for 100 ppm, and 2% Methanol in 0.5% tween 80 + 100 J2 for 200 ppm concentration was taken as control).

^††^ Mean of 4 replications in percent; ^‡^ Arcsine transformation values in parentheses.

***Values in the same column, followed by a common letter, are statistically not different as per Duncan’s multiple range test at 5% level of significance (p<0.05).

**Discussion**

The nematicidal potential of ten *P. lilacinum* and two *Paecilomyces variotii* isolates against *M. incognita* was studied. Our study showed that the 21-day-old culture filtrate of *P. lilacinum* isolates can inhibit the hatching of *M. incognita* eggs up to varying extant. Eggs-hatching inhibition up to 97.55%, 90.52% and 62.97% was obtained with the culture filtrate of Pl 6887 isolate at 100%, 50% and 25% concentrations, respectively. However, in a different study, hatching inhibition was quite lower with the 8-day-old culture filtrate of the same isolate (Rompalli et al. 2016). It suggests that the accumulation of compounds detrimental to nematode egg hatching is greater as the age of fungal culture increases in broth. Similar kind of results were found by dela Piza (2003), where the 30-day-old culture filtrate of *P. lilacinum* completely inhibited *M. incognita* egg-hatching. Similarly, many studies support that the culture filtrate of *P. lilacinum* is effective in inhibiting egg hatching of various root-knot nematode species (Cayrol et al. 1989, Khan and Goswami 2000, Gapasin et al. 2011). Perveen and Shahzad (2013) reported egg-hatching inhibition of *M. incognita* by culture filtrate of *Paecilomyces variotii* isolates. However, it was not better than *P. lilacinum* isolates. In our study, *Paecilomyces variotii* isolates, Pav 1833, showed 90.58%, 56.88%, and 28.74% hatching inhibition at 100, 50 and 25% concentrations, respectively, which was found better than the results obtained with three isolates of *P. lilacinum* (Pl 4483, Pl 5596 and Pl 6948). The culture filtrate of the *P. lilacinum* isolates, especially, Pl 6887 and Pl6553 was found toxic to the *M. incognita* eggs. The eggs were found vacuolated and deformed. Similar morphological changes in the eggs due to exposure to the culture filtrate of *P. lilacinum* are reported earlier (Khan and Goswami 2000).

In our study, we found that the ethyl acetate extract of *P. lilacinum* isolates was toxic to *M. incognita* juveniles. Siddiqui et al. (2000) also earlier proved the higher toxicity of ethyl acetate extract of *P. lilacinum* to *M. javanica* juveniles. The per cent mortality achieved with Pl 6553 isolate was 43.33% and 92% after 48 hours of exposure at 100 and 200 ppm concentrations, respectively. We found a total of seven earlier reported nematicidal compounds from Pl 6553 isolate, including four potent nematicidal fatty acids, namely, lauric, myristic, stearic, and butyric acid, with a per cent area of 0.11, 0.16, 0.22, and 0.43 respectively. Stadler et al. (1994) screened nematicidal activities of fatty acids produced in cultures of two Basidiomycetes viz., *Pleurotus pulmonarius* and *Hericium coralloides*. It was found that the LD50 value of linoleic and myristic acid against *Caenorhabditis elegans* was 5 ppm, 25 ppm for lauric, palmitic and oleic acid, and 50 ppm for stearic acid. Their study highlighted the importance of saturated and unsaturated fatty acid mixtures produced by nematophagous fungi and their contribution to nematicidal activity. Zhang et al. (2012) determined the nematicidal property of fatty acids (butyric, caproic, caprylic, capric, lauric, myristic, palmitic, oleic and linoleic acid) against *M. incognita*. It was found that the fatty acids were effective in inhibiting egg-hatching, killing second-stage juveniles and overall suppression *M. incognita* population, both in *in-vivo* and *in vitro* conditions. However, those fatty acids were not obtained from *P. lilacinum*. Park et al. (2004) identified linoleic acid and oleic acid from cultures of *P. lilacinum* isolates. Production of palmitic, stearic, oleic and linoleic acid was also reported earlier from *P. lilacinum* (Teles and Takahashi 2013, Sharma et al. 2014c). The nematicidal potential of butyric acid was described earlier (Browning et al. 2004, Browning et al. 2006, Ntalli et al. 2010, Zhang et al. 2012), with the majority of the reports suggesting butyric acid is effective against *Meloidogyne* spp. However, there are fewer reports where it was secreted by *P. lilacinum* (Sharma et al. 2014c). Butyric acid was present in four isolates of *P. lilacinum* we studied, namely, Pl 4910 (0.48), Pl 5596 (0.74), Pl 6064 (0.85), and Pl 6553 (0.43).

Our study is the first report on the production of lauric and myristic acid by *P. lilacinum* (isolates Pl 6553 and Pl 6064). However, both lauric and myristic acid were reported earlier from the toxin-producing Basidiomycetes (Stadler et al. 1994). The juvenile mortality achieved with the metabolites of Pl 6553 was highest followed by Pl 6887, and least with Pl 4483. GC-MS analysis showed 7, 5 and 1 nematicidal compounds from these isolates, respectively. The toxic effect of nematicidal compounds secreted by Pl 6553 was visible as the formation of multiple vacuoles inside the dead juveniles. The phenomenon of vacuole formation is termed methuosis (Maltese and Overmeyer 2014). Palmitic acid was found in every isolate studied, except for Pl 5596 and Pl 6553. Whereas, it was the only nematicidal fatty acid found in both the *Paecilomyces variotii* isolates. It may conclude that *Paecilomyces variotii* is a less efficient producer of nematicidal fatty acids contrary to *P. lilacinum*. Acetic acid with a high per cent area (5.32%), was only found in Pl 6887. The nematicidal property of acetic acid is described earlier (Dijan et al. 1991; Seo and Kim 2014).

Sharma et al. (2020) found a fraction of 20 compounds, with 5 major ones, namely, 2-ethyl butyric acid, Phenyl ethyl alcohol, Benzoic acid, Benzene acetic acid and 3,5-Di-t-butyl phenol, which gave 94.6% egg hatching inhibition, and 62% juvenile mortality against *M. incognita*. However, the nematicidal effect of those five compounds was not determined individually. These compounds, except for 2-ethyl butyric acid and 3,5-Di-t-butyl phenol, were also detected in our study from different isolates. Comparatively, the per cent area of some of these compounds was much higher in the isolates of *Paecilomyces variotii* than in *P. lilacinum*. However, the hatching inhibition obtained from the culture filtrate of *P. lilacinum* isolates was comparatively higher than *Paecilomyces variotii* isolates. It suggests that there may be some other effective compounds playing a role in inhibiting egg hatching that need to be identified.

Chemical nematicides are still a top priority among growers for crop protection against nematodes even after the restriction and lack of availability. However, interest in the biological control of nematodes has increased in recent years, and studies suggest natural products can replace the hazardous chemical nematicides (Zhang et al. 2012; Chen and Song 2021). Nematophagous fungi have potential enough to suppress the nematode population in the field, without causing any adverse effects on the environment (Siddiqui and Mahmood 1996). Our study, supported by the previous reports (Anke and Sterner 1997; Park et al. 2004; Li et al. 2007; Stadler et al. 1994; Sharma et al. 2014c), suggests that nematicidal compounds, derived from microbes, can be inexpensive alternatives to the chemical nematicides apart from being eco-friendly and selective.

In conclusion, the isolates of *P. lilacinum* varied in the production of nematicidal compounds and the presence of these compounds in higher numbers plays a significant role in an overall reduction of the root-knot nematode by inhibiting egg-hatching and/or killing juveniles. We found that the culture filtrate of the isolates Pl 6887 and Pl 6553 was highly effective in inhibiting *M. incognita* egg hatching. Along with that, Pl 6553 secreted compounds that are highly detrimental to second-stage juveniles. Our study demonstrates that the presence of nematicidal secondary metabolites in the solvent affects the survival of nematodes, and higher juvenile mortality can be achieved when more nematicidal compounds are present in number. While Pl 6887 is already a commercialized isolate in India, Pl 6553 is a potential candidate for further research and field trials, with its diverse range of nematicidal compounds. The compounds we considered as nematicidal in our study, are the compounds already reported as nematicidal from previous studies. It does not conclude that these are the only nematicidal compounds secreted by the isolates we tested.

**Acknowledgement:** The authors wants to thank Head, Division of Plant Pathology and In-charge, Indian Type Culture Collection, IARI, New Delhi for providing the fungal isolates.

**References**

Abad P, Gouzy J, Aury JM et al (2008) Genome sequence of the metazoan plant-parasitic nematode *Meloidogyne incognita*. Nat Biotechnol 26:909-915. <https://doi.org/10.1038/nbt.1482>

Anke H, Sterner O (1997) Nematicidal metabolites from higher fungi. Curr Org Chem 1(4):361-74.

Browning M, Dawson C, Alm, SR, Gorres JH, Amador JA (2004) Differential effects of butyric acid on nematodes from four trophic groups. Appl Soil Ecol 27(1):47-54. <https://doi.org/10.1016/j.apsoil.2004.03.005>

Browning, M, Wallace DB, Dawson C, Alm SR, Amador JA (2006) Potential of butyric acid for control of soil-borne fungal pathogens and nematodes affecting strawberries. Soil Biol Biochem 38(2):401-404. <https://doi.org/10.1016/j.soilbio.2005.05.020>

Cayrol JC, Djian C, Pijarowski L (1989) Study of the nematicidal properties of the culture filtrate of the nematophagous fungus *Paecilomyces lilacinus*. Revue de Nematologie 12(4):331-336

Chen JX, Song BA (2021) Natural nematicidal active compounds: Recent research progress and outlook. J Integr Agric 20(8):2015-2031. <https://doi.org/10.1016/S2095-3119(21)63617-1>

Degenkolb T, Vilcinskas A (2016) Metabolites from nematophagous fungi and nematicidal natural products from fungi as an alternative for biological control. Part I: metabolites from nematophagous ascomycetes. Appl Microbiol Biotechnol 100:3799-3812. <https://doi.org/10.1007/s00253-015-7233-6>

Degenkolb T, Vilcinskas A (2016) Metabolites from nematophagous fungi and nematicidal natural products from fungi as an alternative for biological control. Part II: metabolites from nematophagous basidiomycetes and non-nematophagous fungi. Appl Microbiol Biotechnol 100:3813-3824. <https://doi.org/10.1007/s00253-015-7234-5>

dela piza RM (2003) Nematicidal activity of *Paecilomyces lilacinus* (Thom) Samson culture filtrate against root-knot nematode, *Meloidogyne incognita* (Kofoid and White) Chitwoood. B.S. Thesis, Leyte State University

Desaeger J, Wram C, Zasada I (2020) New reduced-risk agricultural nematicides-rationale and review. J Nematol 52(1):1-16. <https://doi.org/10.21307/jofnem-2020-091>

Djian C, Pijarowski L, Ponchet M, Arpin N, Favre-Bonvin J (1991) Acetic acid: a selective nematicidal metabolite from culture filtrates of *Paecilomyces lilacinus* (Thom) Samson and *Trichoderma longibrachiatum* Rifai. Nematologica 37(1-4):101-112. <https://doi.org/10.1163/187529291X00105>

Ehwaeti ME, Phillips MS, Trudgill DL (1998) The viability of *Meloidogyne incognita* eggs released from egg masses of different ages using different concentrations of sodium hypochlorite. Nematologica 44(2):207-219. <https://doi.org/10.1163/005325998X00081>

Eisenback JD (2020) *Meloidogyne incognita* (root-knot nematode), Invasive Species Compendium. CABI, Wallingford, UK. <https://www.cabi.org/isc/datasheet/33245>

Gapasin RM, Vasquez EA, Rendon GA (2011) Bioassay-guided identification of the nematicidal secondary metabolites from *Paecilomyces lilicanus* for the control of root-knot nematode (*Meloidogyne graminicola*, Golden and Birchfield). Ann trop res 33(2):22-43. <https://doi.org/10.32945/atr3322.2011>

Hewlett TE, Dickson DW, Mitchell DJ, Kannwischer-Mitchell M (1988) Evaluation of *Paecilomyces lilacinus* as a biocontrol agent of *Meloidogyne javanica* on tobacco. J Nematol 20(4):578

Huang WK, Cui JK, Liu SM, et al (2016) Testing various biocontrol agents against the root-knot nematode (*Meloidogyne incognita*) in cucumber plants identifies a combination of *Syncephalastrum racemosum* and *Paecilomyces lilacinus* as being most effective. Biol Control 92*:*31-37. <https://doi.org/10.1016/j.biocontrol.2015.09.008>

Jatala P (1986) Biological control of plant-parasitic nematodes. Annu Rev Phytopathol 24(1):453-489. <https://doi.org/10.1146/annurev.py.24.090186.002321>

Jones JT, Haegeman, A, Danchin, EG et al (2013) Top 10 plant‐parasitic nematodes in molecular plant pathology. Mol Plant Pathol 14(9):946-961. <https://doi.org/10.1111/mpp.12057>

Kassam R, Yadav J, Chawla G et al (2021) Identification, characterization, and evaluation of nematophagous fungal species of *Arthrobotrys* and *Tolypocladium* for the management of *Meloidogyne incognita*. Front Microbiol 12:790223. <https://doi.org/10.3389/fmicb.2021.790223>

Kesba H, Abdel-Rahman A, Sayed S, Al-Sayed AS (2021) Screening the nematicidal potential of indigenous medicinal plant extracts against *Meloidogyne incognita* under lab. and greenhouse conditions. Egypt J Biol Pest Control 31:1-12. <https://doi.org/10.1186/s41938-021-00429-y>

Khan MR, Goswami BK (2000) Effect of culture filtrates of *Paecilomyces lilacinus* isolates on hatching of *Meloidogyne incognita* eggs. Ann Plant Prot Sci 8(1):62-65

Kiewnick S, Sikora RA (2006) Biological control of the root-knot nematode *Meloidogyne incognita* by *Paecilomyces lilacinus* strain 251. Biol Control 38(2):179-187. <https://doi.org/10.1016/j.biocontrol.2005.12.006>

Luangsa-Ard J, Houbraken J, van Doorn T, Hong SB, Borman AM, Hywel-Jones NL, Samson RA (2011) *Purpureocillium*, a new genus for the medically important *Paecilomyces lilacinus*. FEMS Microbiol Lett 321(2):141-149. <https://doi.org/10.1111/j.1574-6968.2011.02322.x>

Luangsa-Ard JJ, Hywel-Jones NL, Samson RA (2004) The polyphyletic nature of *Paecilomyces* sensu lato based on 18S-generated rDNA phylogeny. Mycologia 96(4):773-780. <https://doi.org/10.1080/15572536.2005.11832925>

Mai WF, Abawi GS (1987) Interactions among root-knot nematodes and Fusarium wilt fungi on host plants. Annu Rev Phytopathol 25(1):317-338. <https://doi.org/10.1146/annurev.py.25.090187.001533>

Maltese WA, Overmeyer JH (2014) Methuosis: nonapoptotic cell death associated with vacuolization of macropinosome and endosome compartments. Am J Pathol 184(6):1630-1642. <https://doi.org/10.1016/j.ajpath.2014.02.028>

Ntalli NG, Vargiu S, Menkissoglu-Spiroudi U, Caboni P (2010) Nematicidal carboxylic acids and aldehydes from Melia azedarach fruits. J Agric Food Chem 58(21):11390-11394. <https://doi.org/10.1021/jf1025345>

Park JO, Hargreaves JR, McConville EJ, Stirling GR, Ghisalberti EL, Sivasithamparam K (2004) Production of leucinostatins and nematicidal activity of Australian isolates of *Paecilomyces lilacinus* (Thom) Samson. Lett Appl Microbiol 38(4):271-276. <https://doi.org/10.1111/j.1472-765X.2004.01488.x>

Peiris PUS, Li Y, Brown P, Xu C (2020) Fungal biocontrol against *Meloidogyne* spp. in agricultural crops: A systematic review and meta-analysis. Biol Control 144*:*104235. <https://doi.org/10.1016/j.biocontrol.2020.104235>

Perveen Z, Shahzad S (2013) A comparative study of the efficacy of *Paecilomyces* species against root-knot nematode *Meloidogyne incognita*. Pak J Nematol 31(2):125-131

Pulavarty A, Egan A, Karpinska A, Horgan K, Kakouli-Duarte T (2021) Plant parasitic nematodes: A review on their behaviour, host interaction, management approaches and their occurrence in two sites in the Republic of Ireland. Plants 10(11):2352. <https://doi.org/10.3390/plants10112352>

Rompalli R, Mehendrakar SR, Venkata PK (2016) Evaluation of potential biocontrol agents on root-knot nematode *Meloidogyne incognita* and wilt causing fungus *Fusarium oxysporum* f. sp. *conglutinans* in vitro. Afr J Biotechnol 15(19):798-805. <https://doi.org/10.5897/AJB2015.15235>

Samson RA (1974) *Paecilomyces* and some allied hyphomycetes. Stud Mycol 6:1-119

Seo Y, Kim YH (2014) Control of *Meloidogyne incognita* using mixtures of organic acids. Plant Pathol J 30(4):450. <https://doi.org/10.5423%2FPPJ.NT.07.2014.0062>

Sharma A, Gupta A, Dalela M, Sharma S, Sayyed RZ, Enshasy HAE, Elsayed EA (2020) Linking organic metabolites as produced by *Purpureocillium Lilacinum* 6029 cultured on Karanja deoiled cake medium for the sustainable management of root-knot nematodes. Sustainability 12(19):8276. <https://doi.org/10.3390/su12198276>

Sharma A, Sharma S, Yadav S, Naik SN (2014) Role of Karanja deoiled cake based medium in production of protease and fatty acids by *Paecilomyces lilacinus* 6029. J Biosci Bioeng 118(3):270-271. <https://doi.org/10.1016/j.jbiosc.2014.02.020>

Siddiqui IA, Qureshi SA, Sultana V, Ehteshamul-Haque S, Ghaffar A (2000) Biological control of root rot-root knot disease complex of tomato. Plant Soil 227:163-169. <https://doi.org/10.1023/A:1026599532684>

Siddiqui ZA, Mahmood I (1996) Biological control of plant parasitic nematodes by fungi: a review. Bioresour Technol 58(3):229-239. <https://doi.org/10.1016/S0960-8524(96)00122-8>

Stadler M, Anke H, Sterner O (1993) Linoleic acid—The nematicidal principle of several nematophagous fungi and its production in trap-forming submerged cultures. Arch Microbiol 160:401-405. <https://doi.org/10.1007/BF00252228>

Stadler M, Mayer A, Anke H, Sterner O (1994) Fatty acids and other compounds with nematicidal activity from cultures of Basidiomycetes. Planta Med 60(2):128-132. <https://doi.org/10.1055/s-2006-959433>

Teles APC, Takahashi JA (2013) Paecilomide, a new acetylcholinesterase inhibitor from *Paecilomyces lilacinus*. Microbiol Res 168(4):204-210. <https://doi.org/10.1016/j.micres.2012.11.007>

Zhang WP, Ruan WB, Deng YY, Gao YB (2012) Potential antagonistic effects of nine natural fatty acids against *Meloidogyne incognita*. J Agric Food Chem 60(46):11631-11637. <https://doi.org/10.1021/jf3036885>
