# Supplementary material for: Chemo-profiling of Purpureocillium lilacinum and Paecilomyces variotii isolates using GC-MS analysis, and evaluation of their metabolites against M. incognita
Source: PLoS One. 2024 Feb 15;19(2):e0297925. doi: 10.1371/journal.pone.0297925 (PMC10868743; doi:10.1371/journal.pone.0297925)
Supplement: Attachment — Submitted filename: Response to Reviewers.docx [file pone.0297925.s015.docx]

**Response to Reviewer #1:**

The comments and revisions are in the attached file. Some issues include:

Query 1-What is the situation of the laboratory for conducting mortality and inhibition of egg for Meloidogyne?

Reply 1. The laboratories where experiments were conducted are in Division of Nematology, and Division of Plant Pathology, ICAR- IARI, New Delhi. The laboratories are well established, well maintained and have all the facilities required to conduct juvenile mortality and hatching inhibition assay. The mass culture of *M. incognita* was developed under glasshouse controlled conditions on tomato. The experiments were conducted under the supervision of experts in the field of nematology and plant pathology.

Query 2-Figure 1 for juvenile of Meloidogyne is lack of quality.

Ans 2. We have included the figures of better quality for juvenile of Meloidogyne.

Query 3-The graphs for GC was not provided.

Reply 3. The GC chromatogram are included in the supporting figure (Fig S1 to Fig S12).

Query 4-The source of Meloidogyne is not clear.
Reply 4. The *Meloidogyne incognita* was obtained from Division of Nematology, ICAR-IARI, New Delhi.

Query 5-The experimental design is not clear
Reply 5. The complete randomized design was followed.

Query 6-The molecular identification of Meloidogyne is not provided.

Reply 6. The molecular identification of *Meloidogyne incognita* with figure is now provided in the revised manuscript*.*

Query 7-Why authors have used Duncan for analysis?

Reply 7. Duncan multiple range test is one of the most common method to compare treatment means, especially when difference are larger among treatments, as our data in hatching inhibition and juvenile mortality assay possesses larger difference in their means. Along with that, DMRT is most commonly used method to compare means in biological experiments. This is the reason we used Duncan for analysis.

**Response to additional Editor Comments:**

Your paper contains such good information; however, it needs a significant revision.

1-The English style must be revised by a professional native English speaker.

**Reply:** We have tried our best to improve English style with English professional.

2-GC result must be provided in material and methods, result and discussion by details.

**Reply:** GC-MS related all information included in material and methods, results and discussion in details.

3-Molecular identification of Meloidogyne incognita must be provided.

**Reply:** The molecular identification of *Meloidogyne incognita* with figure is now provided in the revised manuscript*.*

4-The references must be in line with the PloS One style.

**Reply:** References has been corrected as per Plos One style.
