# Supplementary material for: Chemo-profiling of Purpureocillium lilacinum and Paecilomyces variotii isolates using GC-MS analysis, and evaluation of their metabolites against M. incognita
Source: PLoS One. 2024 Feb 15;19(2):e0297925. doi: 10.1371/journal.pone.0297925 (PMC10868743; doi:10.1371/journal.pone.0297925)
Supplement: Attachment — Submitted filename: Response to Reviewers.docx [file pone.0297925.s016.docx]

**Regarding reference list**

The reference list is reviewed and corrected based on journal requirements. In Table 2, the references were not properly placed in the older manuscript are now corrected with addition of few more references for butyric acid.

**Additional Editor Comments:**

1-The phylogenetic tree, Figure 2, number 0.00 and other with decimal must be deleted from the tree. Keep only percentage. In addition, the percentage must be in the right place, not to cross the lines. Branch length no need to give. The legend for the Figure 2 then accordingly must be corrected.

Response: From the phylogenetic tree, i.e., Fig. 1, number 0.00 and other with decimal are deleted. The legend for the Figure 1 is accordingly corrected.
